# Porcelain Fillings

**Published:** 1904-02

**Authors:** W. S. Capon

**Affiliations:** Philadelphia, Pa.


### PORCELAIN FILLINGS.

### AFTER 12 YEARS.

By W. S. CAPON, D. D. S., Philadelphia, Pa.

It is just twelve years since I made my first porcelain inlay, and having devoted the greater part of my professional career to the practice and study of this class of work I feel that an experience covering so many years on a comparatively new subject should be received as having sufficient value to be a guide to many who have already acquired the first rudiments and a little experience.

Almost daily I am asked the question, "To what extent will I recommend that porcelain should be used?" I reply that much depends on one's adaptability to unite art with mechanics. In other words, to know when and where beauty and strength can be harmonized to a practical extent. Such a knowledge is desirable in general dentistry, but in a great degree requisite in the practice of this special branch.

My first public clinic was given ten years ago, and while I succeeded in interesting a few, even those would shake their heads and turn away saying that cement would wash from the joints and it seemed nonsense to give time to what would likely be inevitable failure.

At each succeeding clinic the interest increased until now a dental society meeting is not complete unless a demonstration of this work is included as an attraction—in fact, many times it is the headline; for even a very indifferent demonstration shows the great possibilities of porcelain..

My operations have extended over many hundreds of cases, and it is to be expected that our best results come from simple inlays. I mean by that term a cavity filled with porcelain on the labial or buccal surfaces of teeth where there is no strain or wear except that of ordinary cleanliness; yet it is a surprise to know the great durability of corner sections.

I have a number of cases ranging from eight to twelve years of service that I have watched with great interest, and in a few cases they have been positively abused, and yet they stand the strain although held with cement without the aid of pins or other means.

My experience is equally satisfactory with central and lateral cross-sections, even to those of lower incisors, which you all know are difficult teeth to repair. I have only had to replace one tip, and that was on a very prominent central, after nearly nine years of use.

Porcelain is an ideal filling in approximal cavities. It saves poor structure and, to a great extent, conceals the fact that they are filled more than in many other positions. It is very gratifying to know that by this means I have been able to give a natural appearance to the teeth of many ladies who came to me as young girls with cement or discolored gutta-percha remnants and a source of satisfaction to these young patients, not to have the same cavities excavated and refilled every few months.

I have been alluding to the six anterior teeth because it is there we find the greater number of opportunities where porcelain is applicable. Next I shall mention the distal approximal surfaces of cuspids, especially when the cavity is extensive. In such places porcelain is an excellent material because it enables the operator to overcome what is a most tiresome operation with gold, in a manner acceptable and gratifying both in restoration and durability. The same can be said of bicuspids; but now I am approaching treacherous ground, because these teeth are much used and more frequently decayed, offering a temptation for an esthetic operation such as can be obtained with a greater degree with this material.

A bicuspid is a small molar presenting large surfaces, and it has large contours with proportionately small supporting surfaces with a closely occluding antagonist that locks with every motion required for mastication. Add to this the great force brought to bear on an underlying substance like porcelain, which is stuck in position, and then you have an idea of the requirements necessary for a successful porcelain filling.

It may seem an exaggeration when I say, the larger the cavity the better the chance for success, and in many cases where I have supplied the whole palatine or buccal surface, they have given the greatest satisfaction, having had to replace but one in all these years. I account for that because of easy accessibility and the opportunity to change the form of the tooth, a better foundation giving the resistance of a large surface without leverage of an overhanging contour.

It is not my desire to discourage the filling of these teeth with porcelain, but rather to give you guidance that may save disappointments. I have had failures, but I am glad to say that my successes more than counterbalance them, otherwise I could not stand before you as an exponent of this work without fear of loss to my professional standing.

Passing from treacherous ground I will just say a few words about putting porcelain in molars and term it dangerous practice, because its use indiscriminately in these teeth has done more to discourage beginners than in any other portion of the mouth. Much that I have said regarding bicuspids can be applied to molars, and yet under a few circumstances porcelain may be used to advantage; but in no case has its durability been equal to that which has been shown in other teeth.

The first objection is the difficulty in procuring sufficient space to obtain a correct mould of the cavity, and even when there are such opportunities, there is still the difficulty in holding the metal rigid until a perfect outline is secured. Succeeding in this there is still some uncertainty, as large surfaces in being fused frequently change form sufficient to cause a poor joint, which is a weak spot through loss of cement with wear and wash. I have been successful in making some very fair molar fillings and they have stood the wear for a number of years, but upon close observation I notice that the joints seem a trifle larger, which is caused by the constant pressure in mastication, breaking minute particles off the edges.

It is more than fourteen years since porcelain was used in a practical manner and as it is at the present time. Since then other methods have been devised, principally for use of a low fusing body. It is not my wish to open this question so much aired during the last two or three years, as to the virtues of low and high fusing porcelain. Both have their defenders and equal results are claimed by each side. I

have always used the high fusing body and my statements are based on the use of that material, and it has a record unapproached by any other of its kind.

Of course, it is to be expected that in twelve years our materials would show improvement. The most notable is the variety of shades in porcelain and the uniformity of fusing, two great assistants. The improvement in appliances is the addition of electricity and the latest furnaces are simply perfect in form and practical working, making the possibility for perfect work within the reach of all.

During this time we have known how to do the work, but its ultimate success depended on one material and that was cement. It was our anchor and our only hope. Its failure meant more than I, for one, care to contemplate, and while it is one of the materials that has not been improved in the life of this work and it is still deficient, yet I forgive it for the total good results. It has spoiled the beauty of our almost perfectly shaded inlay and caused much disappointment, but it has virtues that will probably never be equaled. I have used many that are good and those that had the greatest number of requisites as assistants to porcelain I used loyally and through their efficiency I am enabled to give you this paper.

In the last year or two I have added to my list, and if after a few years I find them equal to my standard I shall give them all the credit due.

Any dentist practicing this branch of dentistry and having fair success will learn many advantages that will compensate for any losses that may arise through the acquirement of the knowledge and practice sufficient to insure confidence because tooth structure is supplied with a material approximating what has been lost. It strengthens poor teeth to a surprising degree—in fact, I have seen many cases where teeth have broken away, leaving the porcelain strong and intact, particularly in bicuspids. It is less tiresome to both patient and operator and allows a brief respite to the unavoidable exchange of vitiated atmosphere to which unfortunately we are subjected in the practice of dentistry. One of its greatest recommendations is the fact of being a perfect non-conductor, at least as near perfect as any filling can be.

Many times it is asked if my experience has not shown that cement irritates the pulp and eventually causes death, particularly in large labial cavities where the teeth are hyper-sensitive. My reply has been, as it is now; my assertion after more matured experience, that I have no such trouble, although I keep a close record of all operations. I advance two reasons for such good results—one theoretical and the other practical. First, it may be that in labial cavities the amount of cement being a very thin layer, the harmful influence is reduced to a minimum, and yet in other cavities I know there is considerable cement between the porcelain and bottom of cavity, so that at that rate this theory would seem valueless. My practical way of reducing a probability of trouble with any filling material is to coat the cavity with rubber lining varnish. I do not always use it with porcelain, because it is not advisable to risk the possibility of destroying the natural affinity that cement has for tooth substance. This might seem like drawing a very fine line, but very thin inlays have small means of retention and every point must be considered.

As I am hovering over debatable ground I may be allowed to make a statement in regard to sensitive teeth prepared for crowns. A large part of my practice of porcelain consists of using to a marked extent the porcelain jacket, which obviates the necessity of killing pulps. In many hundreds of these crowns I do not treat one per cent. for any after trouble of root or portion of tooth crowned. I make this statement for the benefit of all, but especially for those dentists who kill everything in sight or near it. I know that there are men who never cover a tooth in any manner without first devitalizing. If such practice is good dentistry, then I, for egize for the terrible array of living pulps that I have proudly placed to my credit.

To reiterate what I have contended for years and what must now be accepted, porcelain fillings do not fall out through deterioration of cement; they improve with age and they are the best of non-conductors. They are held by tenacity of cement and undercuts in cavities are not necessary, but an undercut in the porcelain is imperative.

It is the strongest and most durable filling for poor teeth, and the limit of its possibilities is regulated by the experience of the operator. It saves time and health and is most desirable to the majority of patients and, as one of my lady patients in poor health recently said, "It is delightfully restful."

Rubber dam is always unpleasant, and with some people it is positively unbearable; with this work the use of it is reduced to rare occasions.

If by accident or improper preparation a filling becomes loose, it may be replaced with little trouble, even after it has been in use for many years, and a close examination will find the cavity perfectly clean with a hardened wall of dentine.

In conclusion, let me ask the prejudiced ones if porcelain has not a right to be placed on the list with the other permanent filling materials, and those who practice this work earned the privilege of being considered at least fair dentists if they do save teeth outside of old prescribed methods.

To the unbiased I would ask a fair consideration, as the practice of porcelain does not interfere with the saving of teeth otherwise, and is a grand assistant.

To those who are favorably impressed and are endeavoring to help with the good work, I will ask of them a continuance of their efforts with the precaution to be careful and not too enthusiastic, so that in time our records will be such that we may have undisputed position in the special work of dental ceramics.

DISCUSSION.

Dr. Joseph Head:—Recent records brought to our knowledge by Dr. Kirk show conclusively that gold was used in the eighth century for filling, and the length of time that it has been used, in addition to all the theories, would conclusively prove to most of us that gold is a very good filling material; yet we must remember that only ten or twelve years ago there was a great outcry against the use of gold, and amalgam, cements and the other fillings for a time bid fair to drive gold from the field, and yet, I think, gentlemen, that gold will still be used in spite of its manifest drawbacks. Yet it is most surprising in this advanced age of scientific research that one of our best filling materials should be a material that has to be applied by the crude method of hammering instead of being cemented. It is most surprising that a patient should have to be subjected to the grave inconvenience of having a non-adhesive material pounded, through a long period of operation into a painful cavity. In spite of the great record that gold has made, we must always remember that there is this grave and serious objection to the ordinary metallic filling, inasmuch as it is held in place simply and purely by means of undercuts, that it is non-adhesive to the tooth structure. It is a scientific axiom that an adhesive filling is far superior to one that is non-adhesive. The adhesive filling will be a tooth preserver from the edge to the very bottom of the cavity, a non-adhesive filling will probably only be a tooth preserver so far as it prevents the entrance of bacteria on the edge, for we have found almost universally that if the cohesive gold was defective on any part of the edge, decay almost universally followed. We know from experience that in cases of cement fillings bacteria may enter, yet the bacteria that do enter find themselves in such an unproductive country that, like an army deprived of its commissariat, they die and are incapable of doing harm. Therefore, in speaking of the porcelain inlay in contradistinction to all unadhesive fillings materials, I would say that, while the perfectly adapted inlay preserves tooth structure partially by excluding bacteria and has the great advantage that it may match the tooth, it also has the property of inhibiting the growth of bacteria that may enter the margins.

Dr. Osmun:—It has been said that dentists get a particular thing that proves successful, in their hands, and exclude all other kinds of operations forthwith. They take one particular kind of gold, one particular kind of enamel, or one favorite kind of cement and stick to that. They get in the habit of using porcelain, they have success with it, and from that day on they are porcelain users only. All kinds and conditions of fillings must be porcelain. Another man, who has failures with it, condemns it, and falls back on gold. While I am delighted to hear Dr. Capon's paper, it seems to me that all these different fillings have their merits, and the question of their use is one of judgment. What do you, gentlemen, as individual operators, think? If one says porcelain fillings are of no use, why does he say so? The keynote is that the dentist must use an educated judgment so as to know when to use these various and different methods. I have had some experience with porcelain fillings that has been exceedingly satisfactory, and some that I wish I had not had. I have had some experience with gold fillings that was very satisfactory indeed, and some that was not so satisfactory—the other fellow has done them over after I got through! But I do not condemn gold, neither do I condemn porcelain. Neither do I tie up to porcelain fillings, and say that there is nothing else but that with which to fill teeth. I try to select my filling material according to the case in hand, and where we can do that we will find that all these various methods and kinds of fillings will be of advantage to us and a blessing to our patients.

Dr. Duffield:—There is one point in Dr. Capon's paper which I think requires a great deal of consideration from the society, and that is the statement that he rarely, if ever, devitalizes teeth, prior to capping. There is no doubt in my mind that all teeth that are devitalized, no matter how carefully the work may be done, are susceptible to future trouble which seldom occurs in teeth containing live pulps.

Dr. Chas. A. Meeker:—I have used porcelain fillings and commenced with glass fillings some fifteen or twenty years ago. When I first saw Dr. Jenkins do his work with porcelain, it seemed so very easy that I thought I could do it. That is the trouble with the American dentist—he can do anything! I put in quite a number of fillings, and, as time passed on, took them out and put in something else. All dentists have to buy their experience. Possibly I use more judgment now than I did then when I put in a porcelain filling. I have owned, I think, eight different furnaces. I have one large McBriar furnace now, and I use it in making crowns and inlays. Where I find that the principle of mechanics will support such a filling, I use an inlay, and my success is reasonably good. I have had some in now for four years. I saw one a few days ago which, four years ago, I placed in a central incisor, and it was doing good work. I have seen many others that have been in a year or a year and a half where the patients have not been very particular in the care of the teeth, and the fillings are all doing good work.

Dr. Richards:—This is a very interesting subject, and I think it would be wise, if those who think that by and by they will adopt it, to take this opportunity of asking questions concerning this method, as we have with us two disciples in the art.

I would like to ask Dr. Capon or Dr. Head what method they use in regard to the buccal cavity on the lower second molar or wisdom tooth; how it is kept dry underneath the margin of the gum?

Dr. Chase:—Dr. Duffield has touched on the question of the devitalizing of the pulp and the essayist also referred to it. I very rarely find it necessary so to do; I do not believe in the wholesale destruction of pulps; if it becomes necessary, as it sometimes does, well enough, but to simply devitalize the pulp for the purpose of making a crown, I most decidedly condemn and do not practice it, and stand on the same ground as Dr. Capon.

Dr. Pruden:—I would like to ask Dr. Capon how he removes porcelain inlays? I had very great difficulty with a case recently, and I should like to ask that question.

Dr. Sanger:—I can say one or two things from my own experience with porcelain inlays which may possibly be of some slight value.

One thing is as to the question of color. Following a paper read by Dr. Head last winter, in New York, I took up a line of experiments to overcome, if possible, the effect of light, from different directions, on porcelain inlays. You who have put in porcelain inlays know that the great bugbear of the operator is the fact that when you look directly at the inlay the color may be all right, but when you look at it from one side the color is all wrong; from different angles we get different effects, according to the transparency of the materials used. I have succeeded in overcoming this difficulty slightly by using as the basis of the inlay a dead white color, a color which is almost absolutely opaque, and then laying over the shade which I desire to produce, following out in a measure, the principle that is applied in the making of porcelain teeth in the trade, where the biscuit is dense first, and afterwards the shades are produced by laying over the first body, as I understand it.

This is only a suggestion, for you who are porcelain workers must meet that difficulty.

Dr. Capon:—I think that a paper going over a term of ten or twelve years is more acceptable to you, than one which only covers four or five years, because I think time has something to do with the position.

I want, too, to rectify a misunderstanding under which some seem to be laboring, by saying that I do not wish and never desired to antagonize the use of gold at any time. I merely suggest porcelain in the light of a strong recruit in a company of volunteer soldiers. Move along a little bit and let the new man in; he is strong and able to take his part. I do not deprecate the use of gold in its place, but porcelain has its place also, and I am delighted to know that it is accepted today by those who have had experience in that line.

I do not believe it is possible to make an opaque body translucent; the nearest we can come to it is to fuse thoroughly and trust to our cement.

Many of you have never seen porcelain inlays of longer standing than six or eight years, but I would like you to see one that Dr. ———— has after ten years' use in the mouth. I have no work here, but Dr. Head can show you some that I put in three or four years ago, and I have some in the mouth of Dr. Stokes, of Philadelphia, which will show the idea both for gold caps and inlay work.

I do not always use inlays; sometimes, if I cannot get a proper mould I leave them alone entirely.

The rubber lining that I refer to is only a fine solution of rubber varnish with two or three other ingredients, and is very thin, and I do not always use it.

Concerning the question asked with reference to the removal of an inlay, I never remove an inlay and try to put it back again, because the inlay is always broken; I never took one out but that the edges were broken or it was completely destroyed. Many times I have removed them where one part has been broken entirely away, and left the other part there; in such instances it is a case of burs vs. porcelain. I always make a new inlay, and never expect to take one out to be returned because the cavity will not be the same; you must cut a great deal, and with a great deal of strength.

Regarding the strength of inlays and their density and I have had cases where I have cut the corner sections of inlays down to the finest edge and not broken them off.

